# The Cultivable Bacteria Colonizing Canine Vagina During Proestrus and Estrus: A Large-Scale Retrospective Study of Influencing Factors

**DOI:** 10.3390/ani14233460

**Published:** 2024-11-29

**Authors:** Sabine Schäfer-Somi, Dominik Lechner, Alexander Tichy, Joachim Spergser

**Affiliations:** 1 Clinical Center for Reproduction, University of Veterinary Medicine Vienna, 1210 Vienna, Austria; dominik@lechner.vet; 2 Institute for Bioinformatics and Biostatistics, University of Veterinary Medicine Vienna, 1210 Vienna, Austria; alexander.tichy@vetmeduni.ac.at; 3 Center for Pathobiology, University of Veterinary Medicine Vienna, 1210 Vienna, Austria; joachim.spergser@vetmeduni.ac.at

**Keywords:** dogs, vagina, bacteria, mycoplasma, estrus

## Abstract

Background: The evaluation of canine vaginal bacteria during proestrus and estrus and the chosen parameters will help to prevent the misuse of antibiotics in veterinary medicine. Methods: This study carried out a retrospective analysis to obtain microbiological examination results for vaginal swabs from 696 healthy dogs, all of whom were patients from one clinic. Age, body weight, fur length (long or short hair), the time of sampling of vaginal swabs, the duration of proestrus/estrus, progesterone (P4) concentration (the first and last samples), and bacteriological findings were evaluated. Results: Only 3.7% (26/696) of the samples were negative for bacteria. The impact of fur length was low. However, the calculation of the odds ratios revealed that the chance for a high-grade growth of *Escherichia* (*E.*) *coli* and *Mycoplasma* (*M.*) *canis* increases with body weight class. The earlier the sampling was performed, the lower the number of high-grade cultures (*p* < 0.01). The duration between the first and last measurement of progesterone (P4) was positively related to the cultural detection of *Pasteurellaceae*, *Streptococcus* spp., and *Enterococcus* spp. (*p* < 0.05) and negatively to the cultivation of *Mycoplasma* spp. (*p* < 0.01). Conclusions: Our findings reflect some likely factors contributing to the variability of the physiological vaginal flora in dogs, which may help interpret cultured bacteriological findings.

## Simple Summary

To determine the best time for mating, many breeders will ask for bacteriological examinations of their dog’s vagina. The results of these examinations are variable, but in many cases an antibacterial therapy will be initiated in a clinically healthy dog. Therefore, we aimed to investigate the physiological canine vaginal flora during proestrus and estrus, and we retrospectively evaluated bacteriological findings from 696 healthy breeding dogs, all of whom were patients from a single clinic. Furthermore, the bacteriological findings were related to possible influencing factors like age, body weight class, fur length, the time of sampling of vaginal swabs, and the duration of proestrus/estrus. Despite insight into the physiological range of vaginal bacteria, we found that the chance for a high-grade growth of *Escherichia* (*E.*) *coli* and *Mycoplasma* (*M.*) *canis* increases with body weight. Furthermore, the earlier the sampling was performed, the lower the number of high-grade cultures (*p* < 0.01). Additionally, the duration between the first and last measurement of progesterone was positively related to the cultural detection of *Pasteurellaceae*, *Streptococcus* spp., and *Enterococcus* spp. (*p* < 0.05) and negatively to the cultivation of *Mycoplasma* spp. (*p* < 0.01). These findings, in relation to the course of the estrus cycle, may enhance the interpretation of cultured bacteriological findings and help to prevent the misuse of antibiotics in veterinary practice.

## 1. Introduction

Determining the best timing for mating in dogs is a common routine procedure used to increase breeding success [1,2,3]. To evaluate the best time for mating, a vaginal smear for estrus cycle evaluation [4,5,6,7] is taken, and often, an additional sample for microbiological examination is collected since some researchers suspect that certain bacteria may have a pathological effect on fertility [8]; however, others disagree [4,9]. Antibiotic treatment, with or without susceptibility testing, is still a common practice aimed at increasing fertility [5]. However, it is well known that the uncritical, prophylactic use of broad-spectrum antibiotics in breeding dogs leads to an increase in antimicrobial resistance [10,11]. Studying the physiological vaginal microbiota of healthy female dogs will help prevent the misuse of antibiotics in veterinary medicine.

In female healthy dogs, the most common bacteria found in the vagina using culture-based techniques are *Pasteurellaceae*, beta-hemolytic streptococci, *Escherichia* (*E.*) *coli*, *Enterococcus* spp., and coagulase-positive staphylococci [12,13,14,15,16,17,18]. Mycoplasmas and ureaplasmas are likely underestimated by laboratories because they do not test for this group of bacteria, which requires rich culture media for growth and typically needs a week or longer for cultivation [19]. However, *Mycoplasma* (*M.*) *canis* and *Ureaplasma* (*U.*) *canigenitalium* have been isolated from the vagina of healthy bitches and can be considered part of the autochthonous microbiota, even though they may become pathogenic under certain circumstances [5,8,9,13,20,21]. This physiological mixed flora is not believed to compromise fertility [4]; however, in some female dogs, the number of bacteria increases to above-average values, and sometimes, high-grade monocultures can be found in healthy dogs. In a recent retrospective study, 23.254 vaginal swabs were evaluated that had been sent in from different clinics for conventional aerobic bacterial cultivation to three commercial laboratories [22]. In that study, the previous findings were confirmed, and a broad spectrum of both mixed cultures and monocultures was detected in both healthy and diseased dogs. Unfortunately, the cycle stage at the sampling time was not given, and the samples were not examined for mycoplasmas.

The vaginal microbiota of canines undergoes physiological changes during proestrus and estrus [23], likely because of changes in mucin secretion, pH levels, redox potential, bloody secretion, and estrogen concentrations [5,17,24,25,26]. During anestrus, bacteria are rarely found, while in estrus, increased numbers of bacteria can be found [16,23]. However, the spectrum of bacteria in healthy bitches during anestrus is similar to that during proestrus and estrus, mainly comprising *Streptococcus* spp., *Staphylococcus* spp., *M. canis*, and *E. coli* [9]. Only recently, by using 16-S sequencing and DNA extraction, was the vaginal microbiota shown to be larger, and significant changes between proestrus and estrus concerning beta-diversity were found [26]. The composition of the vaginal microbiota not only varies with the cycle stage [5,23,26] but also with the site of sampling, since bacteria were more frequently isolated from the caudal than from the anterior vagina [24]. In one study, increasing age was found to coincide with a change in the vaginal microbiota [27]. Sterilization was not found to have a significant effect on the vaginal flora [28]. The impact of the breed has not been proven so far; only one study found *E. coli* and beta-hemolytic streptococci more frequently in Great Danes and Beagles than in Poodles [5]. However, only genetics were considered and not the impact of body weight, length of the vagina, age, or further factors. The height and length of the vestibule and the length of the vagina are larger with increasing dog weight [29], while vaginal length and width are significantly lower in older than in middle-aged dogs [30]. Between 2018 and 2020, retrospective studies showed the possibility of fur length influencing the cultivable vaginal bacteria of bitches during proestrus and estrus [12,31,32]. These studies suggested that shorter fur is a risk factor, whereas the size of the dog was not found to be relevant. The conclusion indicates that the fur might function as a mechanical barrier, lacking in short-haired dogs; however, the authors acknowledged that additional data would be necessary for a definitive conclusion.

Therefore, in this study, we retrospectively evaluated a broad panel of data following sample size calculation using the program G*Power, and a representative population of healthy breeding dogs in proestrus and estrus was included; all data were derived from patients of our clinic. The aim of this study was to determine the cultivable bacteria colonizing the canine vagina in proestrus and estrus and whether factors like age influence the microbial composition, the time of sampling, the duration of estrus, weight class, and fur length.

## 2. Materials and Methods

### 2.1. Animals and Cases

In this study, we analyzed cases of female dogs examined for breeding timing at the clinic for gynecology, andrology, and obstetrics at Vetmeduni Vienna between 2003 and 2022. For statistical analysis, only healthy and anatomically normal bitches were included. A vaginal sample for microbiological examination, one for vaginal cytology, and at least two blood samples for progesterone (P4) measurements were taken.

For all these cases, the following data were captured: breed, age, body weight (assigned to three weight categories: <10 kg, 10–30 kg, and >30 kg), fur length (grouped as either long or short hair), the time of sampling, the duration of estrus, progesterone (P4) values (the first and last samples), and bacteria-inclusive mycoplasmas isolated from vaginal samples (specification and quantification). All breeding dogs had a body mass index within the normal range and underwent a clinical health examination, which included a gynecological examination involving vaginal cytology and an examination of the mammary glands. Dogs exhibiting inflammatory changes in the vestibule and vagina were excluded from this study.

### 2.2. Sampling and Analyses

Blood sampling for progesterone measurement was carried out from the cephalic vein, and serum was gathered via centrifugation at 3000× *g* for 10 min. All analyses were performed in the Central Laboratory of the Vetmeduni Vienna (A) using a chemiluminescence assay (Cobas^®^, Roche Diagnostics, Mannheim, Germany, with a detection range of 0.64–127 nmol/L of serum). Serum LH concentration was not measured, but the LH peak was estimated to occur when a certain progesterone value was reached, as described in the literature (before LH peak: P4 < 3 nmol/L [33], between LH peak and the beginning of ovulation: ≥3 to <14 nmol/L, [33]; beginning, during, or after ovulation: P4 ≥ 14 nmol/L, [33]).

Samples for microbiological examination were taken in a standardized manner, as all patients were from the same clinic and all veterinarians used the same procedure; thus, the consistency of the sampling procedure was assured. A sterile vaginoscope was inserted as deep as possible into the anterior vagina; then, a sterile swab (Transwab^®^; Medical Wire, Corsham, UK) was inserted through the vaginoscope, rotated gently several times, placed into the culture medium, and either immediately delivered to the laboratory or after a maximum storage of 3 days at +4 °C (over the weekend). All samples were examined at the Institute of Microbiology of the Vetmeduni Vienna (A) as described previously [34]. Briefly, the swabs were plated onto Columbia agar III with 5% sheep blood and MacConkey II agar (BD Diagnostics, Vienna, Austria) using the three-phase streaking method. The plates were incubated aerobically (Columbia agar, MacConkey agar) or anaerobically (Columbia agar) at 37 °C for 48 h. Microbial growth was semi-quantitatively graded as sporadic (<5 colony-forming units (CFUs)), low (5–30 CFUs), moderate (31–100 CFUs), or high (>100 CFUs), depending on the presence and number of colonies in the streaking sections. Bacterial isolates were identified at the species level using biochemical tests (before 2016) or via matrix-assisted laser desorption ionization-time of flight (MALDI-ToF) mass spectrometry (from 2016 onwards), as previously described [35].

To isolate the mycoplasmas and ureaplasmas, the swab samples were placed into a 1 mL 2 SP medium and vortexed; then, 100 µL of suspension was plated onto SP4 agar and Ureaplasma agar, followed by incubation at 37 °C under a 6% CO_2_ atmosphere for up to 7 days. The agar plates were examined daily for the presence of mycoplasma and ureaplasma colonies. For semi-quantification, mycoplasma and ureaplasma colonies were counted, and their growth was graded as sporadic (<5 CFUs), low (5–30 CFUs), moderate (31–100 CFUs), or high (>100 CFUs). Mycoplasma isolates were identified using 16S-23S rDNA PCR-RFLP analysis (before 2016) [15], or through MALDI-ToF mass spectrometry (from 2016 onwards) [35]. Ureaplasma isolates (*U. canigenitalium*) were identified based on their characteristic colony morphology and urease activity.

### 2.3. Statistical Analyses

A G*Power analysis was used to determine the number of cases required for significant conclusions. All the analyses were calculated using IBM SPSS statistics version 29 (IBM Corp., Armonk, NY, USA). Pearson’s chi-square test was used to analyze the relationship between bacterial growth, weight classes, and hair length. The odds ratio (OR) was also calculated from the cross tables. Spearman’s rank correlation coefficient was used to explore the relationship between bacterial findings and other parameters. An analysis for significant differences between groups was performed using the Mann–Whitney test. Values were given as percentages or mean ± SD. A *p*-value below 5% (*p* < 0.05) was considered statistically significant.

## 3. Results

A total of 696 cases were evaluated; 253 dogs were short-haired, and 442 were long-haired. In one mongrel, fur length was not documented.

There were 103 different breeds examined; the most frequent breeds were the Labrador Retriever (n = 95), Golden Retriever (n = 79), Australian Shepherd (n = 23), English Cocker Spaniel (n = 23), Border Collie (n = 21), American Staffordshire Terrier (n = 21), German Shepherd Dog (n = 21), Belgium Shepherd Dog (n = 18), Bernese Mountain Dogs (n = 18), Cavalier King Charles Spaniel (n = 16), and English Setter (n = 15). The mean age of the dogs was 4.4 ± 1.7 years. The distribution among weight classes was as follows: <10 kg (n = 79): 11.3%, 10–30 kg (n = 402): 57.8%, >30 kg (n = 215): 30.9%. Evaluation of the interval between vaginal swab sampling and the first progesterone measurement showed an average of 3.6 ± 3.4 days (0–21 days). The serum concentration of the first progesterone (P4) measurement was, on average, 5.9 ± 10.0 nmol/L (0.64–123.6 nmol/L). In 339 cases, the swabs were taken before the LH peak (P4 < 3 nmol/L) [33] and in 289 cases, they were taken between the LH peak and the beginning of ovulation (≥3 to <14 nmol/L) [33]. In 63 cases, the samples were taken at the beginning of, during, or after ovulation (P4 ≥ 14 nmol/L) [33].

### 3.1. Bacteriological Findings

Only 3.7% (26/696) of the samples yielded negative results (no bacterial growth). Bacterial isolates derived from 696 microbiological examinations were assigned to different groups of bacteria based on taxonomic classification, cultural characteristics, or their prevalence on canine mucous membranes (Table 1).

**Figure 1:**
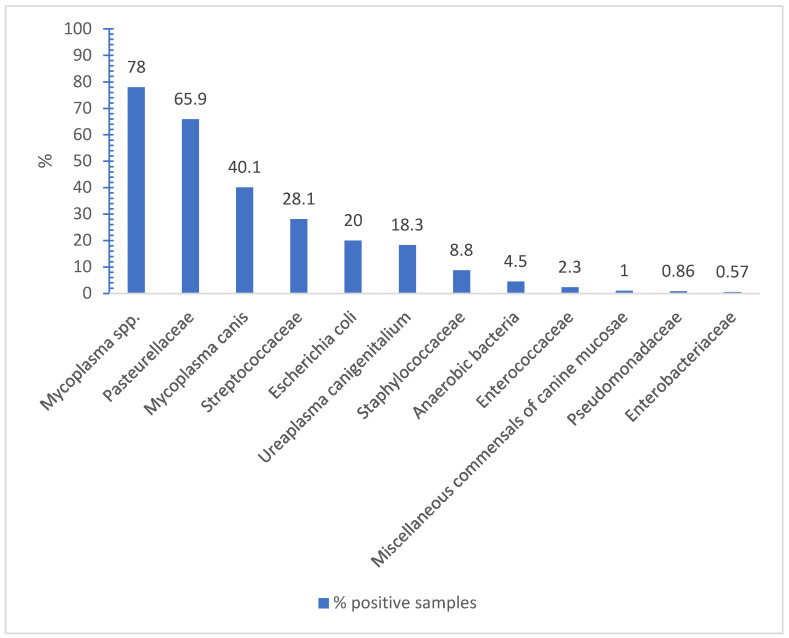
Distribution of isolated bacteria (n = 696 swabs). This figure shows the percentages of samples positive for the respective isolated bacteria and visualizes the distribution of these bacteria within the physiological vaginal flora of healthy female dogs in proestrus and estrus.

Microbiological examinations mostly resulted in mixed cultures with the low-grade to high-grade presence of (with decreasing frequency) *Mycoplasma* spp., *M. canis*, *Pasteurellaceae*, *Streptococcaceae*, *E. coli*, *U. canigenitalium*, and *Staphylococcaceae.* Anaerobic bacteria, *Enterococcaceae*, *Lactobacillus* spp., *Neisseria* spp., and *Pseudomonadaceae* were rarely isolated. A detailed list of all the isolated species is given in Table 1. *Mycoplasma* spp. and *M. canis* were most frequently isolated (78% and 40.1%, resp). The distribution and percentages are shown in Figure 1.

**Figure 2:**
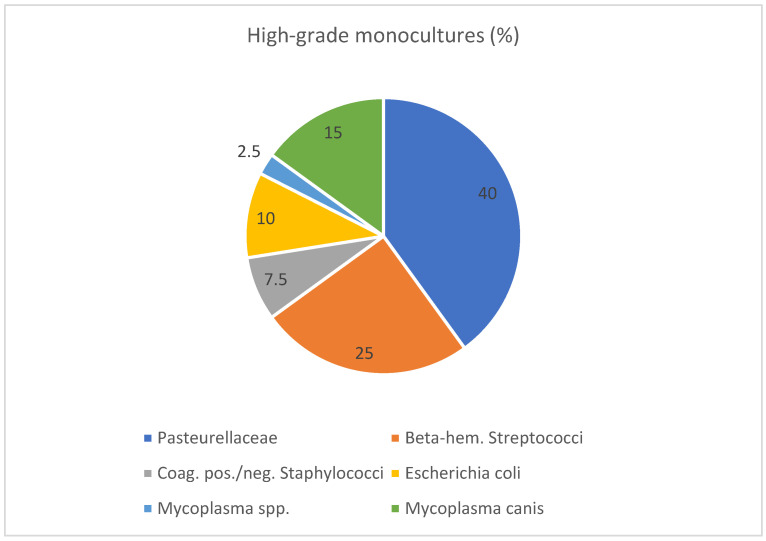
Distribution of high-grade monocultures (n = 40). This figure shows the percentages of different bacteria within the group of high-grade monocultures. Beta-hem. = Beta-hemolytic; Coag. = coagulase; neg. = negative; pos. = positive; spp. = species.

High-grade monocultures only occurred in 40 cases (5.7%, Figure 2) and were predominantly identified as *Pasteurella multocida* and *Canicola haemoglobinophilus*, followed by beta-hemolytic streptococci and *M. canis.* Furthermore, with decreasing frequency, monocultures of *E. coli*, staphylococci, and *Mycoplasma* spp. were found. High-grade mixed cultures were detected in 59.6% of samples.

#### 3.1.1. Hair Length

The cultural detection of *Pasteurellaceae*, beta-hemolytic streptococci, staphylococci, *Enterococcus* spp., *E. coli*, and anaerobic bacteria did not differ significantly between groups with short and long hair. High-grade monocultures of bacteria were isolated in 4.7% of short-haired dogs and 6.3% of long-haired dogs (*p* > 0.05). In both long- and short-haired dogs, mycoplasmas were detected; however, there was a significantly higher prevalence of high-grade *Mycoplasma* spp. growth in short-haired dogs compared to long-haired dogs (*p* < 0.05). *Mycoplasma canis* was isolated in 44.2% of short-haired dogs and 37.7% of long-haired dogs (*p* > 0.05); *U. canigenitalium* was isolated in 20.9% of short-haired dogs and 21.2% of long-haired dogs (*p* > 0.05).

#### 3.1.2. Body Weight

There were no significant differences in the percentages of samples with low-, moderate-, and high-grade growth of *Pasteurellaceae*, beta-hemolytic streptococci, staphylococci, *Enterococcus* spp., anaerobic bacteria, and *Mycoplasma* spp. across different weight classes. However, *E. coli* with high-grade growth was isolated significantly more often in the largest dogs (>30 kg, *p* = 0.004). Dogs with a body weight of >30 kg have a higher likelihood of testing positive for high-grade *E. coli* (OR 1.25) than dogs weighing 10–30 kg or lower (OR 0.28 and 0.22); the likelihood of smaller dogs testing positive is significantly lower. Spearman’s correlation revealed a significant positive relation between body weight and positive *E. coli* culture (*p* = 0.006). High-grade monocultures were rarely found, particularly in the smallest body weight group (*p* < 0.05). The likelihood of isolating a high-grade monoculture is greater in dogs weighing < 10 kg than those weighing 10–30 kg or more (OR 3.10 and OR 1.71, respectively). *Mycoplasma canis* was isolated in all body weight groups in mixed cultures; however, significantly, it was observed more frequently in the >30 kg body weight group (*p* = 0.002). High-grade monocultures of *M. canis* were isolated less frequently and without difference between groups. *U. canigenitalium* was seldom isolated in healthy breeding dogs (<10 kg: 14.3%, 10–30 kg: 20.5%, >30 kg: 15.5%, *p* > 0.05).

#### 3.1.3. Further Parameters

The bacteriological findings were not affected by age; there was no increase in the percentages of high-grade cultures, monocultures, and high-grade monocultures with increasing age, and there was no difference between age groups (1–2 years, n = 32; >2–5 years, 243, n = 429; >5 years, n = 23).

However, the time of sampling (the time between swab sampling and first P4 measurement) was negatively related to the number of high-grade cultures; the earlier the sampling was conducted, the lower the number of high-grade cultivations of beta-hemolytic streptococci, *Pasteurellaceae*, *Mycoplasma* spp., anaerobic bacteria (*p* < 0.05) and *E. coli* (*p* < 0.01). The number increased towards estrus (*p* < 0.01) but did not apply to high-grade monocultures. The duration between the first and last measurement of P4 (5.3 ± 3.0 days, 0–21 days) was positively related to the frequency of *Pasteurellaceae*, beta-hemolytic streptococci, and *Enterococcus* spp. (*p* < 0.05) and negatively related to the frequency of *Mycoplasma* spp. (*p* < 0.01).

## 4. Discussion

It is common practice to perform a microbiological examination of the vagina during the first few days of proestrus in female dogs to determine the best mating time. Antibiotic treatment, with or without susceptibility testing of bacterial isolates, is frequently used to increase fertility [5]. Meanwhile, several studies show that the uncritical, prophylactic use of broad-spectrum antibiotics in breeding dogs leads to an increase in antimicrobial resistance and, among others, to selection of methicillin-resistant *Staphylococcus pseudintermedius* (MRSP) [4,10,11,36]. Unfortunately, breeders are unaware of these consequences; they do not realize the risk of resistant bacteria transmission between dogs and between surroundings and dogs. However, due to growing knowledge and awareness among veterinarians and authorities, a transboundary initiative for improved antibiotic stewardship is currently underway, and official recommendations for using antibiotics in veterinary medicine have been released by the European Medicine Agency [37]. The World Health Organization founded an Advisory Group on Integrated Surveillance of Antimicrobial Resistance (WHO-AGISAR). To prevent the abuse and misuse of antibiotics and to demonstrate that many bacteriological findings do not require treatment, the study of the physiological vaginal flora and microbiota was an important cornerstone.

Several studies with a reasonably representative number of samples show the broad variety of vaginal bacteria that can be isolated in healthy female dogs [26,38]; the most frequently isolated bacteria were confirmed in our study. However, using 16-S sequencing and DNA extraction, more species were detected; notably, Fusobacterium, *Porphyromas*, *Parvimonas*, and *Escherichia-Shigella* were the predominant genera in vaginal samples in a recent study involving 10 female dogs [26]. These species were not differentiated in this study using culture methods and MALDI-TOFF SD. Also, mycoplasmas and even ureaplasmas were detected in healthy female dogs [13,17,21,25,26]. In our study, 78% of samples were positive for *Mycoplasma* spp., 40.1% for *M. canis*, and 18.3% for *U. canigenitalium*. *Mycoplasma canis* was the most commonly isolated species in our representative pool, which is in agreement with other studies [8,21]. In some studies, the percentages were even higher (42–81%, [5,8,39]). In a former study, the *Mycoplasma* species present was shown to not vary between cycle stages [21]; in our study, we demonstrated that the cultural isolation of *M. canis* is related to the duration of the cycle, the presence/amount of other bacteria, and body weight. Apart from *M. canis*, we further identified *M. maculosum*, *M. spumans*, *M. edwardii*, and, very seldomly, *M. cynos*, *M. arginini*, and *M. opalescens* as part of the normal vaginal microbiota in healthy female dogs.

However, little is known about influencing factors on the vaginal flora; while some female dogs repeatedly demonstrate low numbers of bacteria, in others, high-grade mixed cultures and monocultures predominate. This study evaluated factors that might have an impact. All dogs were thoroughly examined vaginoscopically and using vaginal cytology. All were healthy; therefore, diseases are not believed to play a role, as was shown in the case of oral diseases and the oral microbiota [40]. One important finding during this large-scale data evaluation was that the impact of hair length is less important than previously thought [12,31,32]. In former studies, up to 349 samples were evaluated, and the researchers found significantly fewer high-grade cultures in dogs with long hair (*p* < 0.05); this is verified in our study but only for *Mycoplasma* spp. In recent studies [12,31,32], the initial hypothesis was that long fur may contribute to the contamination of the vestibulum and vagina, but it may, on the contrary, have a protective function. However, confounding factors like age and body weight must be considered, and, using a larger population, we found that the high-grade growth of *Mycoplasma* spp. occurred more frequently in short-haired dogs, although these dogs did not show any symptoms of diseases. Unfortunately, no other studies from the literature were available to compare our results; however, the significant number of cases suggests a low rating of the influence of hair length. The site of sampling was of no concern in this study, as all the samples were taken at the clinic and from the anterior vagina using the same method without fail, as occasional sampling from the caudal vagina could falsify the results [24]. Concerning the cycle stage, it is well known that the composition of the vaginal flora changes towards estrus [5,23], which is supposedly related to changing estrogen concentrations [26]. In a recent study, using advanced isolation methods, it was shown that the beta-diversity of the vaginal microbiota mainly changed between cycle stages; between proestrus and estrus, five genera of bacteria, namely *Prevotella*, *Variovorax*, *Porphyromonas*, *Rheinheimera* and *Corynebacterium*, differed significantly. However, these results are based on a small sample of 10 female dogs [26]. In this study, we showed, with many more data, that the earlier the sampling was performed, the lower the number of high-grade cultures of isolates, whereas an increase in duration between the first and last measurement of P4 was positively related to the frequency of certain bacteria but negatively related to the frequency of *Mycoplasma* spp. This indicates, on the one hand, the importance of considering the length of the cycle and the time of sampling and, on the other hand, contemplating mycoplasmas as being part of the normal vaginal flora. In most cases, these bacteria are present in low numbers within the mixed vaginal microbiota. Increasing numbers of other bacteria help to prevent high-grade mycoplasma monocultures; in our study, these bacteria were *Pasteurellaceae*, beta-hemolytic streptococci, and *Enterococcus* spp. In one study, *Streptococcus* spp. was negatively related to uterine infections, and the authors suggested a protective competitive role against more pathogenic bacteria [4].

In one study, increasing age was found to coincide with a change in the vaginal microbiota [27]. The authors evaluated female Beagles between the ages of 1 and 2 years (n = 8), between 3 and 5 years (n = 5), and between 6 and 7 years (n = 10). They found no difference in bacterial species abundance between the groups; however, species diversity changed with increasing age, which was significant between the young dogs (1–2 years) and elderly dogs (6–7 years). Most interestingly, the authors found a change in the metabolism of bacteria with increasing age. However, even though 16S-rRNA gene high-throughput sequencing was used, the number of samples was a limiting factor of the study; in this study, using far more data, we detected no correlation between age and the distribution or number of bacteria. However, to investigate whether the results of Hu et al. [27] are true for a larger population, we decided to choose the same groups of ages as in their study and compared these parameters between groups in this study. It was shown that there was no difference between groups concerning the number and distribution of bacteria, which might be explained by the fact that most dogs belonged to the medium-aged group (>2–5 years). The impact of older age warrants further investigations.

The impact of body weight and length of the vagina were not yet investigated. Only during a pilot study with relatively low patient numbers were these parameters considered [12]; the author stated that body weight was without impact on the distribution and number of vaginal bacteria. However, since the length of the vagina increases with increasing dog weight [29], we wanted to re-evaluate the impact of this parameter. By including 696 bacteriological findings, we finally found that the incidence of high-grade growth of *Escherichia coli* (mixed and monoculture) was highest in the middle and large dog groups (*p* < 0.01). However, in the smallest dog group, the chi-square test revealed significantly more other high-grade monocultures than in the larger group. Interpreting these results is difficult and should be carried out cautiously. While a longer vagina may be a reason for the more frequent isolation of *E. coli* due to impeded self-cleaning, the higher frequency of high-grade monocultures in small dogs cannot be explained. Complex mechanisms involving genetics, metabolism, immune system, feeding, surroundings, and more confounding factors require further investigations with more homogenous groups. Furthermore, the number of dogs with high-grade *E. coli* and other high-grade monocultures was relatively small. Therefore, at present, we can only state that the chance for a high-grade *E. coli* and *M. canis* culture increases with body weight.

In our study, microbiological examinations were carried out prior to mating using culture-based techniques. Therefore, we only detected live bacteria able to grow on artificial media in vitro. Nevertheless, more advanced techniques will not replace culture-based techniques in veterinary praxis in the foreseeable future, even though the latter only detects about 10% of all bacteria in the endometrium and vagina; in healthy dogs in proestrus and estrus, these are mostly derived from the vagina [38]. Therefore, the usefulness of a routine vaginal bacteriological examination in healthy female dogs in proestrus and estrus may be doubted. This only allows for observing changes in the vaginal bacteria composition and antimicrobial resistance over time. We finally emphasize that it is not recommendable to treat dogs with positive bacterial culture results with antibiotics when clinical signs of a disease are absent [4,5]. A positive bacterial culture, even in the case of a high-grade monoculture, is not proof of subclinical infection.

## 5. Conclusions

The strengths of our study are the large number of dogs examined and sampled in one clinic but living in different housings and receiving different diets, and the standardized sampling compared to previous studies. Furthermore, the quantification and specification of mycoplasmas were also strengths. Even though we did not observe the same individual during proestrus and estrus, the large pool enabled us to obtain some insight into the physiological range and change in vaginal bacteria during the cycle, considering selected influencing factors. The limiting factor is the clinical and culture-based approach; however, these findings concerning the course of the estrus cycle may help us to interpret culture-based bacteriological findings and avoid the misuse of antibiotics in veterinary practice.

Future prospective investigations should focus on individual bitches and long-term observations, including further factors like genetics, environment, and lifestyle, and might consider the individual composition of the vaginal secretion.

## Figures and Tables

**Table 1:** Bacterial species were isolated and grouped for statistical analysis.

Group	Species
*Escherichia coli*	*Escherichia* (*E.*) *coli*
*Enterobacteriaceae* *	*Citrobacter* spp., *Citrobacter koseri*, *Klebsiella* spp., *Klebsiella oxytoca*
*Streptococcaceae*	*Streptococcus* (*Sc*.) spp. (non- or alpha-hemolytic) beta-hemolytic streptococci (mostly *Sc. canis*, occasionally *Sc. equi* ssp. *zooepidemicus* and *Sc. dysgalactiae* ssp. *equisimilis*), *Sc. lutetiensis*, *Sc. minor*
*Staphylococcaceae*	*Staphylococcus* (*St*.) spp. *St. pseudintermedius. St. aureus*, *St*. *capitis*, *St*. *hominis*, *Macrococcus caseolyticus*
*Pasteurellaceae*	*Pasteurella* spp., *Pasteurella multocida*, *Canicola haemoglobinophilus*
Miscellaneous commensals of canine mucosae	*Aerococcus* spp., *Bacillus* spp., *Carnobacterium* spp., *Gemella* spp., *Lactobacillus* spp., *Lactococcus* spp., *Myroides* spp., *Neisseria* spp., *Weissella* spp.
*Pseudomonadaceae*	*Pseudomonas* spp., *Pseudomonas aeruginosa*
*Enterococcaceae*	*Enterococcus* (*Ec*.) spp., *Ec. faecalis Ec. canintestini*
Anaerobic bacteria	*Bacteroides pyogenes*, *Clostridium* spp., *Clostridium perfringens*, *Peptostreptococcus canis*
*Mycoplasma* spp. **	*Mycoplasma* (*M.*) *arginini*, *M. cynos*, *M*. *edwardii*, *M. maculosum*, *M. opalescens*, *M. spumans*
*Mycoplasma canis*	*M. canis*
*Ureaplasma canigenitalium*	*U. canigenitalium*

* Except *E. coli*, ** except *M. canis.*

## Data Availability

The data are available upon request from the authors.
